# Melphalan modifies the bone microenvironment by enhancing osteoclast formation

**DOI:** 10.18632/oncotarget.19152

**Published:** 2017-07-10

**Authors:** Ryan C. Chai, Michelle M. McDonald, Rachael L. Terry, Nataša Kovačić, Jenny M. Down, Jessica A. Pettitt, Sindhu T. Mohanty, Shruti Shah, Gholamreza Haffari, Jiake Xu, Matthew T. Gillespie, Michael J. Rogers, John T. Price, Peter I. Croucher, Julian M.W. Quinn

**Affiliations:** ^1^ Bone Biology Division, Garvan Institute of Medical Research, Darlinghurst, Australia; ^2^ Department of Anatomy, University of Zagreb, School of Medicine, Zagreb, Croatia; ^3^ Bone Biology Group, Department of Human Metabolism, Medical School, University of Sheffield, Sheffield, United Kingdom; ^4^ Faculty of Information Technology, Monash University, Clayton, Australia; ^5^ School of Pathology and Laboratory Medicine, The University of Western Australia, Nedlands, Australia; ^6^ Faculty of Medicine and Health Sciences, Monash University, Clayton, Australia; ^7^ College of Health and Biomedicine, Victoria University, St Albans, Australia; ^8^ Australian Institute for Musculoskeletal Science (AIMSS), University of Melbourne, Victoria University and Western Health, St. Albans, Australia; ^9^ St Vincent's Clinical School, Faculty of Medicine, University of New South Wales, Sydney, Australia; ^10^ School of Biotechnology and Biomolecular Sciences, University of New South Wales, Sydney, Australia

**Keywords:** osteoclast, chemotherapy, bone loss, cell stress, bone microenvironment

## Abstract

Melphalan is a cytotoxic chemotherapy used to treat patients with multiple myeloma (MM). Bone resorption by osteoclasts, by remodeling the bone surface, can reactivate dormant MM cells held in the endosteal niche to promote tumor development. Dormant MM cells can be reactivated after melphalan treatment; however, it is unclear whether melphalan treatment increases osteoclast formation to modify the endosteal niche.

Melphalan treatment of mice for 14 days decreased bone volume and the endosteal bone surface, and this was associated with increases in osteoclast numbers. Bone marrow cells (BMC) from melphalan-treated mice formed more osteoclasts than BMCs from vehicle-treated mice, suggesting that osteoclast progenitors were increased. Melphalan also increased osteoclast formation in BMCs and RAW264.7 cells *in vitro*, which was prevented with the cell stress response (CSR) inhibitor KNK437. Melphalan also increased expression of the osteoclast regulator the microphthalmia-associated transcription factor (MITF), but not nuclear factor of activated T cells 1 (NFATc1). Melphalan increased expression of MITF-dependent cell fusion factors, dendritic cell-specific transmembrane protein (Dc-stamp) and osteoclast-stimulatory transmembrane protein (Oc-stamp) and increased cell fusion. Expression of osteoclast stimulator receptor activator of NFκB ligand (RANKL) was unaffected by melphalan treatment.

These data suggest that melphalan stimulates osteoclast formation by increasing osteoclast progenitor recruitment and differentiation in a CSR-dependent manner. Melphalan-induced osteoclast formation is associated with bone loss and reduced endosteal bone surface. As well as affecting bone structure this may contribute to dormant tumor cell activation, which has implications for how melphalan is used to treat patients with MM.

## INTRODUCTION

The alkylating drug melphalan in combination with corticosteroids has been the first-line therapy for patients with multiple myeloma (MM), an incurable plasma cell cancer [1], for many decades. MM is the second most common hematological cancer and its progression results in immunodeficiency, renal failure and osteolytic bone disease [2, 3]. Whilst novel targeted agents such as thalidomide, bortezomib and lenalidomide have been introduced, melphalan remains a commonly used therapy for non-transplant patients and is also used in high-dose therapy associated with autologous stem cell transplant [1]. Although these treatments improve survival, patients typically undergo relapse and the disease progresses. Thus, MM remains largely incurable.

In the early stages of MM development, as with other cancers that metastasize to bone, colonizing cancer cells localize to specialized niches where they reside in a dormant or low proliferating state for long periods of time [4]. Recent studies have shown that in MM cells of the osteoblast lineage play an important role in the endosteal niche and have the capacity to hold MM in a dormant state for a prolonged period [5, 6]. In this state, dormant cells are resistant to conventional chemotherapies, including melphalan, that target proliferating tumor cells and are available to re-populate the tumor following treatment withdrawal [4, 5]. We have recently shown that osteoclasts, the cells responsible for normal bone resorption, can remodel the endosteal niche and re-activate dormant tumor cells [5]. Osteoclasts therefore play a role in both controlling the re-activation of dormant MM cells and in mediating the development of osteolytic MM bone disease.

Osteoclasts are multinucleated bone-resorbing cells derived from hematopoietic progenitors in the bone marrow under the control of RANKL produced by local osteoblast lineage cells [7]. RANKL can bind its cognate receptor RANK and activate signaling molecules needed for osteoclast differentiation, including the transcription factors NFκB, NFATc1 and MITF. MM cells also produce RANKL and induce its production in the local bone microenvironment, thereby increasing osteoclastic bone resorption and promoting bone loss [8–12]. This tumor-associated bone loss can be mitigated by anti-osteoclastic therapies such as bisphosphonates, or anti-tumor therapies such as melphalan. However, while melphalan treatment *in vivo* kills proliferating tumor cells, it also results in activation of dormant myeloma cells [5], which may have implications for disease progression. This observation might be explained by increased osteoclast resorption and if so would have implications for both tumor relapse and the risk of bone fractures. However, the effect of melphalan treatment on osteoclasts is unknown. Indeed, given the toxic effects of melphalan on bone marrow cell populations, which contain osteoclast progenitors, a decrease in osteoclastic resorption would be expected. However, given the significant implications of pro-osteoclastic effects we investigated the effect of melphalan treatment on the endosteal niche in bone. We found that melphalan treatment causes rapid bone loss in mice and strongly enhances osteoclast formation through several mechanisms.

## RESULTS

### Melphalan increases osteoclast numbers and decreases bone volume *in vivo*

**Figure 1:**
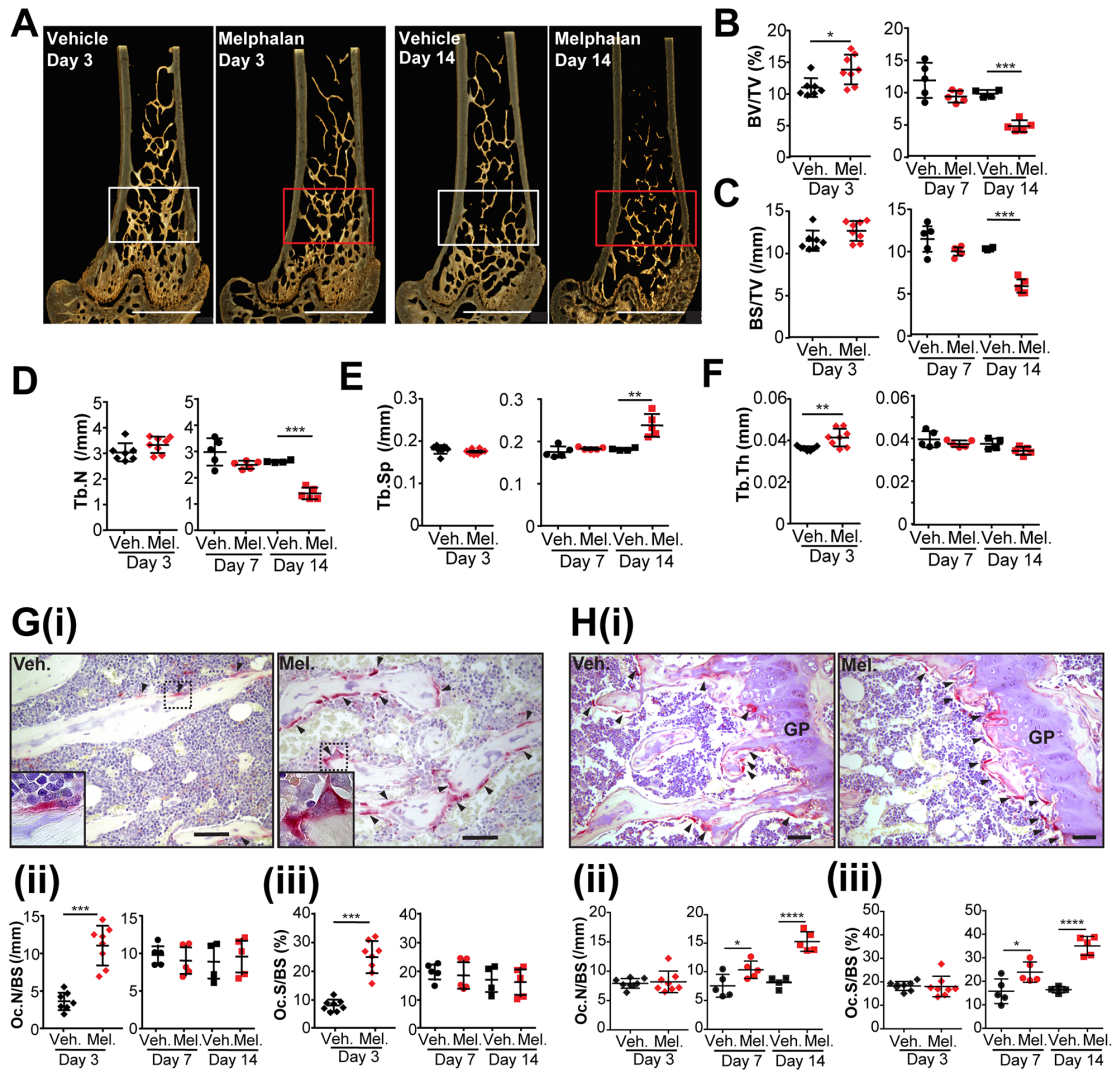
Melphalan treatment increases osteoclast numbers in bone and reduces bone mass in mice **(A)** Three-dimensional reconstructions of microCT scans of representative femora of mice treated with vehicle or melphalan for 3 and 14 days as indicated; regions of interest for analysis are indicated by white or red box; scale bar = 1mm. **(B-F)** MicroCT analysis of the trabecular bone in the secondary spongiosa of the femora of mice treated with vehicle (Veh.) or melphalan (Mel.) for 3, 7 and 14 days, including **(B)** trabecular bone volume/tissue volume (BV/TV) **(C)** trabecular bone surface area/tissue volume (BS/TV), **(D)** trabecular number (Tb.N), **(E)** trabecular separation (Tb.Sp) and **(F)** trabecular thickness (Tb.Th). **(G)** Histological analysis of osteoclasts in the femoral secondary spongiosa showing (i) hematoxylin stained histological sections of the femora with arrowheads identifying TRAP^+^ osteoclasts (red color) on trabecular bone surfaces (scale bar = 100μm; high power view of boxed area shown bottom left) and quantification by histomorphometry of (ii) osteoclast numbers and (iii) bone surface covered by osteoclasts. **(H)** Histological analysis of osteoclasts in the femoral growth plate and primary spongiosa showing (i) hematoxylin stained sections of the femora with arrowheads identifying TRAP^+^ osteoclasts (GP = growth plate, scale bar = 50μm); and quantification of (ii) osteoclast numbers and (iii) bone surface covered by osteoclasts. Numerical data presented as individual data points with indicated mean ± SD and represent 4-8 mice per group. *p<0.05; **p<0.01; ***p<0.001 using ANOVA/Dunnett's *post hoc* test or (for two-way comparisons) Student's t-test.

8-week old male mice were treated with melphalan for 3, 7 and 14 days. Microcomputed tomography (microCT) analysis of the metaphyseal region of the distal femur showed melphalan had little or no significant effects at days 3 and 7, but after 14 days treated mice showed reduced trabecular bone volume (51%) and bone surface (43%) compared to controls (Figure 1A, 1B, 1C). This reduced bone mass was associated with lower trabecular number (46%, Figure 1D) and higher trabecular separation (33%, Figure 1E) with unchanged trabecular thickness (Figure 1F). Melphalan also significantly reduced trabecular bone volume and surface at the growth plate and primary spongiosa at day 14 (Supplementary Figure 1A-1C). This was associated with a decrease in trabecular number (Supplementary Figure 1D) and increase in trabecular separation (Supplementary Figure 1E) with trabecular thickness unchanged (Supplementary Figure 1F). Melphalan did not affect the cortical bone (data not shown).

Histomorphometric analysis of trabecular bone in the distal femoral bone metaphysis demonstrated spatial differences in osteoclast response to melphalan treatment. The number of osteoclasts and the osteoclast surface per bone surface within the trabecular bone (the secondary spongiosa) were increased at day 3 compared with vehicle-treated mice (Figure 1G). This was not seen at later time points (Figure 1G, ii and G, iii). In contrast, within the growth plate and primary spongiosa, we observed an increase in the number of osteoclasts and osteoclast surface per bone surface after 7 and 14 days of melphalan treatment (Figure 1H). This was consistent with the significant reduction in trabecular bone volume (Figure 1A–1F) and primary spongiosa (Supplementary Figure 1) observed at day 14.

Interestingly, a transient increase in trabecular bone volume and thickness was observed after 3 days of melphalan treatment (Figure 1B, 1F). This is likely explained by the increase in osteoblast numbers at this time-point (Supplementary Figure 2A) reflecting the coupling to increased bone resorption at this time [13]. Osteoblast numbers were otherwise unaffected at later time-points.

### Melphalan treatment increases osteoclast progenitor number *in vivo*

**Figure 2:**
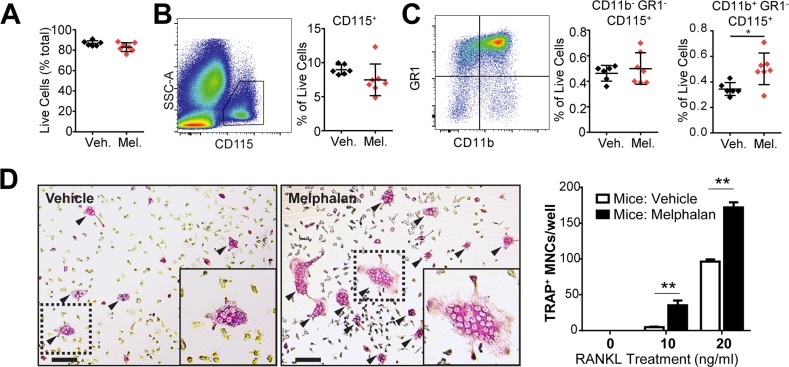
Melphalan treatment *in vivo* results in altered osteoclast progenitor numbers in the femoral bone marrow **(A-C)** Flow cytometry analysis of bone marrow cells from mice treated with vehicle (Veh.) or melphalan (Mel.) for 3 days. **(A)** Total live cells were analyzed as a proportion of all cells. Live cells were then analyzed to determine relative numbers of **(B)** monocytes/macrophages (CD115^+^), **(C)** immature macrophage subpopulations (CD11b^−^/GR1^−^/CD115^+^) and more mature macrophages (CD11b^+^/Gr1^−^/CD115^+^). **(D)** Bone marrow cells from mice treated with vehicle or melphalan were assessed for osteoclast formation *ex vivo* in the presence of 25ng/ml M-CSF in combination with 0, 10 and 20ng/ml RANKL for 6 days; scale bar = 50μm. For graphs in (B) and (C) data is presented as individual data points with indicated mean ± SD. *p<0.05; **p<0.01; ***p<0.001 using Student's t test (n = 8). Graph in (D) shows mean and S.E.M. using ANOVA/Dunnett's *post hoc* test (n = 8).

Bone marrow cell populations are altered by cytotoxic agent treatment, we therefore examined the effect of melphalan treatment on bone marrow cells by flow cytometry. Melphalan did not affect total cell numbers (Figure 2A, Supplementary Figure 3A) although B-cells were decreased by melphalan treatment (Supplementary Figure 3B), consistent with its effects on myeloma cells. The numbers of granulocytes were increased (Supplementary Figure 3B) as well as dendritic cells (Supplementary Figure 3C). Total monocyte/macrophage lineage cell numbers (CD115^+^) were unchanged by melphalan (Figure 2B); immature macrophage populations (CD11b^−^ GR1^−^ CD115^+^) were also unchanged (Figure 2C left), but mature osteoclast/macrophage lineage (CD11b^+^ GR1^−^ CD115^+^) cells were significantly increased (Figure 2C right). To investigate whether melphalan treatment affects osteoclast progenitor numbers in the bone marrow, we measured osteoclast formation from BMCs *in vitro*, when stimulated with M-CSF and RANKL. Indeed, BMCs from melphalan-treated mice yielded much greater osteoclast numbers than those from vehicle-treated mice (Figure 2D). No osteoclast formation was observed in the absence of RANKL.

### Melphalan treatment directly enhances osteoclast formation *in vitro* and increases levels of both MITF protein and MITF-dependent expression of cell fusion-related genes

**Figure 3:**
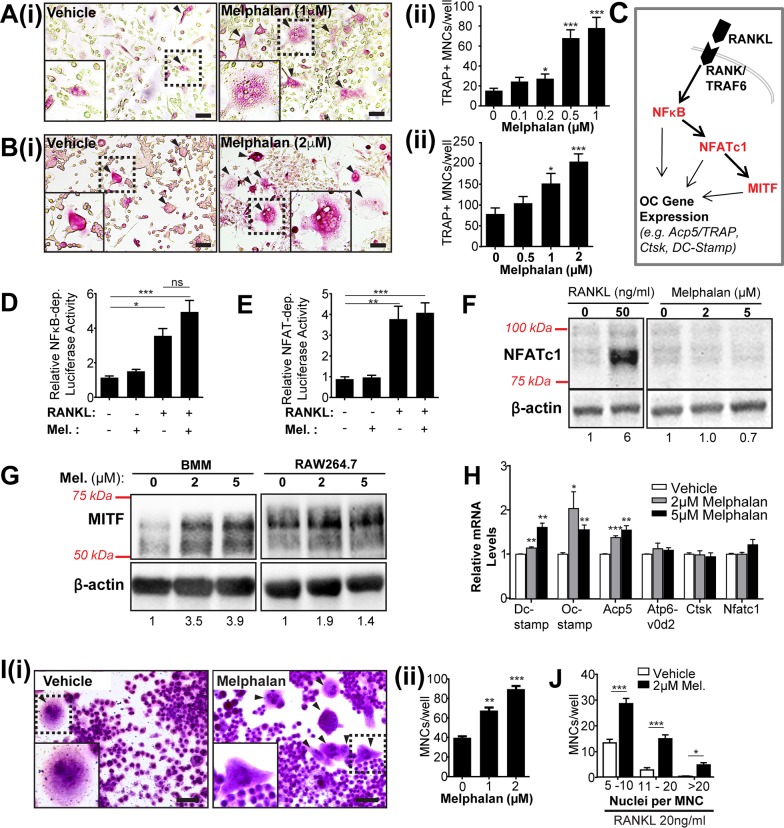
Melphalan treatment *in vitro* enhances osteoclast formation and increases MITF but not NFATc1 levels in osteoclast progenitor populations **(A)** Histochemical analysis of TRAP^+^ multinucleated cells (MNCs) of bone marrow cells cultured in 20ng/ml RANKL and 25ng/ml M-CSF in combination with doses of melphalan or vehicle for 6 days. (i) Photomicrographs showing histochemical staining of TRAP^+^ MNCs indicated by arrowheads (boxed area shown at higher magnification in inset image) and (ii) quantitative dose response data from cultures (n = 4). Scale bars = 50 μm. **(B)** Histochemical analysis of TRAP^+^ MNCs of RAW264.7 cells cultured in 20ng/ml RANKL with melphalan or vehicle for 6 days (i) Photomicrographs showing histochemical staining of TRAP^+^ MNCs indicated by arrowheads and (ii) quantitative dose response data from cultures (n = 4). Scale bars = 50 μm. **(C)** Simplified diagram of transcription factor induction cascade induced by RANKL/RANK binding causing increased levels of active NFkB, NFATc1 and MITF. Note many important factors in this cascade (e.g., AP-1 and p38) are omitted for clarity. RANKL and melphalan treatment for 24 h on the induction of signals in **(D)** NFkB-RAW and **(E)** NFAT-RAW reporter cells, showing induction by RANKL but not by melphalan. **(F)** Western blot analysis of NFATc1 protein levels in RAW264.7 cells treated with RANKL, vehicle or melphalan for 48 h. **(G)** Western blot analysis of MITF protein levels of bone marrow macrophages (BMMs) and RAW264.7 cells treated with vehicle or melphalan in the absence of RANKL for 48 h. **(H)** qRT-PCR-analysis of osteoclast-associated mRNA levels in RAW264.7 cells treated with vehicle or melphalan in the absence of RANKL for 24 h. **(I)** Histochemical analysis of MNCs formed in RAW264.7 cells treated with vehicle or melphalan in the absence of RANKL for 6 days. (i) Photomicrographs of MNCs as indicated by arrowheads and (ii) quantification of the MNCs. Scale bars = 50μm. **(J)** Quantification of TRAP^+^ MNCs in RAW264.7 cells treated with 2μM melphalan in the presence of RANKL for 6 days. In photomicrographs (A, B and I) the boxed areas shown at higher magnification in inset images. Quantitative data from *in vitro* experiments presented as mean ± S.E.M. from 4 independent experiments. *p<0.05, **p<0.01, ***p<0.001, using ANOVA/Dunnett's *post hoc* test.

To determine whether melphalan affects osteoclast progenitors as they undergo differentiation, RANKL- and M-CSF-treated BMCs were cultured with melphalan (or vehicle) for 6 days. Melphalan dose dependently increased osteoclast formation (Figure 3A). In RANKL-stimulated RAW264.7 cell cultures, melphalan similarly enhanced osteoclast formation with maximal effects at 2 μM (Figure 3B). Higher concentrations of melphalan caused extensive cell death (data not shown). Osteoblast mineralization was unaffected *in vitro* by melphalan treatment (Supplementary Figure 2B).

To investigate the mechanisms that underlie melphalan enhancement of osteoclast formation, we assessed its effects on key RANKL-stimulated pathways required for differentiation, including NFκB, NFATc1 and MITF (Figure 3C). NF-κB- and NFAT-RAW reporter cells responded strongly to RANKL stimulation but not to melphalan treatment (Figure 3D, 3E), nor did melphalan enhance the RANKL-elicited signals. Western blot studies also confirmed that melphalan had no effect on NFATc1 levels (Figure 3F), indicating NFκB and NFATc1 nuclear factors are unlikely to mediate melphalan effects on osteoclast formation. In contrast, MITF protein levels in bone marrow macrophages (BMMs) and RAW264.7 cells showed an increase with melphalan treatment at 2 and 5 μM (Figure 3G), suggesting that the enhanced RANKL-elicited osteoclast formation occurs at least partly through MITF-dependent pathways.

To examine this, the effect of melphalan on osteoclast-associated (and RANKL/MITF dependent) gene expression was measured in RAW264.7 cells. After melphalan treatment (2 and 5 μM) for 24 hours, mRNA was extracted and analyzed by quantitative RT-PCR (qRT-PCR). Expression of tartrate resistant acid phosphatase (TRAP; *Acp5*) mRNA was increased by melphalan treatment 1.5 fold (Figure 3H) as was that of osteoclast cell fusion factors *Dc-stamp* and *Oc-stamp*, although not vATPase V0d2 subunit (*Atp6v0d2*) (Figure 3H). mRNA expression of Cathepsin-K (*Ctsk*) and *Nfatc1* were not affected. The effects on *Dc-stamp* and *Oc-stamp* mRNA by melphalan implicated cell fusion effects, which we investigated with RAW264.7 cells. These (but not BMM or bone marrow cells) have a natural propensity to fuse in culture to form macrophage polykaryons, even in the absence of RANKL. Melphalan treatment increased multinucleated cell (MNC) numbers (and nuclear number) in RAW264.7 cultures in the absence of RANKL (Figure 3I). In the presence of RANKL, melphalan also markedly increased the numbers of large osteoclasts with high nuclear numbers (Figure 3J).

### Melphalan actions on osteoclasts show cell stress dependence

**Figure 4:**
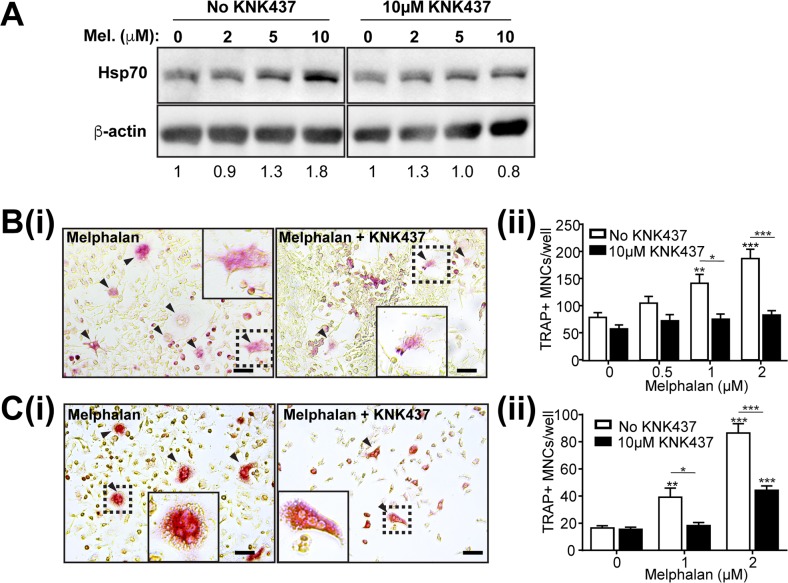
Enhancement of osteoclast formation caused by melphalan is reduced by HSP inhibitor KNK437 **(A)** Western blot analysis of Hsp70 protein levels in RAW264.7 cells treated with melphalan in the absence and presence of HSP inhibitor KNK437 for 48 h. **(B)** Histochemical analysis of TRAP^+^ MNCs in RAW264.7 cells treated with RANKL and melphalan in the absence and presence of KNK437 for 6 days. Scale bars = 50 μm (i) Photomicrographs of TRAP^+^ MNCs as indicated by arrowheads and (ii) quantitative data showing the effects of KNK437 on TRAP^+^ MNCs formed in RANKL and melphalan and RANKL treated cultures of RAW264.7 cells. **(C)** Histochemical analysis of TRAP^+^ MNCs in bone marrow cells treated with M-CSF, RANKL and melphalan in the absence and presence of KNK437 for 6 days. (i) Photomicrographs of TRAP^+^ MNCs as indicated by arrowheads and (ii) quantitative data showing the effects of KNK437 on TRAP^+^ MNCs formed in RANKL and melphalan treated cultures. In photomicrographs (B and C) the boxed areas shown at higher magnification in inset images. Scale bars = 50 μm. Data presented as mean ±S.E.M. from 4 independent experiments. *p<0.05, **p<0.01, ***p<0.001 relative to vehicle treated controls, using ANOVA/Dunnett's *post hoc* test.

Increased osteoclast differentiation and higher MITF levels elicited by melphalan is consistent with cell stress response (CSR)-induced effects on osteoclasts [14, 15]. Indeed, melphalan treatment increased protein levels of the CSR marker heat shock protein 70 (Hsp70) (Figure 4A, left), an action reduced by co-administration of the HSP inhibitor KNK437 (Figure 4A, right). Consistent with this and our earlier observations [14], melphalan effects on osteoclast formation in RAW264.7 cells were greatly reduced by KNK437 (Figure 4B), which, was also seen in BMC cultures (Figure 4C). Notably, KNK437 treatment itself did not affect RANKL-dependent osteoclast formation in the absence of melphalan.

### Melphalan effects on local RANKL expression are weak or absent

**Figure 5:**
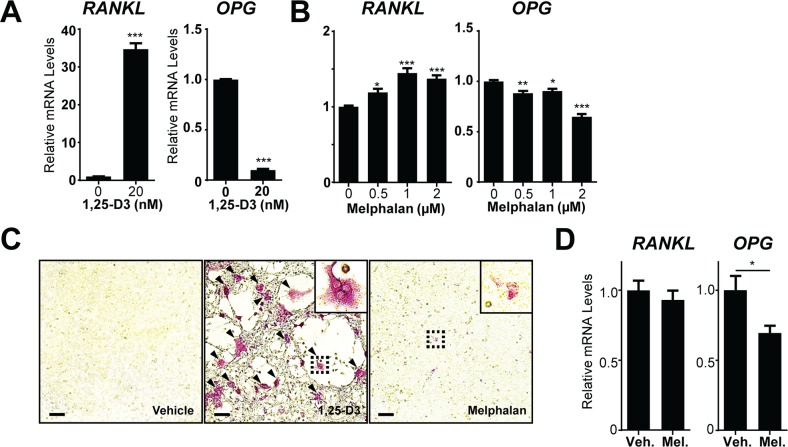
Melphalan increases RANKL expression in primary bone marrow stromal cells (BMSCs) *in vitro* qRT-PCR analysis of RANKL and OPG mRNA in BMSC treated with **(A)** 1,25-D3 or **(B)** melphalan for 24 h (n = 3).**(C)** Histochemical analysis of TRAP^+^ MNCs in co-cultures of fresh bone marrow cells and *in vitro* expanded BMSCs treated with vehicle, 1,25-D3 or melphalan for 6 days (n = 3). Scale bars = 50μm. **(D)** RANKL and OPG mRNA levels were determined in whole bones (tibiae) of mice treated for 3 days with melphalan or vehicle (n = 8). Data presented as mean ±S.E.M. *p<0.05, **p<0.01, ***p<0.001 relative to vehicle treated controls, using ANOVA/Dunnett's *post hoc* test or t-test for 2-way comparison.

To address the possibility that melphalan may affect osteoclast activity *in vivo* by stimulating local levels of RANKL, we assessed melphalan effects on mRNA expression of RANKL and OPG (the secreted decoy receptor of RANKL) in bone marrow stromal cells (BMSCs). These populations contain mature and immature osteoblasts and related cells that strongly express RANKL in response to osteolytic stimuli such as 1,25-D3. Indeed, 1,25-D3 treatment increased BMSC RANKL mRNA levels 36 fold and decreased OPG mRNA by over 90% (Figure 5A). Melphalan treatment increased RANKL and decreased OPG mRNA levels but only very modestly in comparison (Figure 5B). We therefore tested whether melphalan-treated BMC/BMSC co-cultures support osteoclast formation. Positive controls (1,25-D3 stimulation for 7 days) showed large-scale osteoclast formation in these co-cultures, but melphalan treatment resulted in only occasional TRAP^+^ mononuclear cells (Figure 5C) and no osteoclast formation. Furthermore, no increase in RANKL mRNA was observed in bones of mice treated with melphalan over 3 days (Figure 5D). This suggests that melphalan affects osteoclast formation mainly through its effect on osteoclast-intrinsic factors rather than increasing RANKL levels.

## DISCUSSION

In this study we have found strong evidence that melphalan therapy modifies the bone microenvironment by stimulating the formation of osteoclasts. Treatment of tumor-naïve mice with melphalan caused loss of more than half the trabecular bone over two weeks [5]. This was mediated by an increase in osteoclast numbers caused by melphalan treatment. Although some cytotoxic drugs affect bone formation by suppressing gonadal hormone levels [16], melphalan did not reduce osteoblast numbers. Furthermore, the bone loss was associated with lower trabecular number (rather than trabecular thickness), which is a hallmark of osteoclast-mediated bone loss. Whilst these studies were performed in young mice and myeloma typically occurs in older individuals, these data provide important evidence that melphalan treatment increases osteoclast formation and causes bone loss.

Melphalan treatment strongly increased osteoclast numbers on mature trabecular bone surface by day 3 (Figure 1G), which is consistent with the pro-osteoclastic effects of melphalan seen *in vitro* and suggests melphalan is a potentially osteolytic stimulus with the potential to disrupt cell surface niches such as those containing dormant tumor cells [5]. Whilst osteoclast numbers were not increased in this region at later time points they were elevated in the growth plate region, which explains the decrease in bone surface and bone volume.

Our *in vitro* investigations found strong mechanistic evidence that melphalan stimulates osteoclast progenitor differentiation, mainly by enhancing their responsiveness to RANKL. RANKL drives both normal and pathophysiological osteoclast formation [17] but melphalan caused little or no increase in RANKL levels in bone or osteoblasts. In contrast, melphalan had a number of effects on osteoclast progenitors. Firstly, mouse bone marrow formed more osteoclasts *ex vivo* suggesting that more osteoclast progenitors are present; this was supported by the increased CD11b^+^ cell numbers in bone marrow. Secondly, melphalan very strikingly and directly increased osteoclast formation in RANKL-treated progenitors. We studied cell stress in melphalan treated osteoclastic cultures and found that melphalan induced a CSR, similar to previous findings with heat shock protein 90 (HSP90) inhibitors [14, 15, 18]. Presumably, unlike HSP90 inhibitors, this was due to cytotoxicity (e.g., protein misfolding) rather than direct activation of heat shock factor 1 (HSF1), the key transcription factor for CSR. CSR blockade by KNK437 rescued the stimulating effect on osteoclasts without affecting baseline RANKL-elicited osteoclast formation. MITF protein levels, critical for osteoclast gene expression, were also elevated by melphalan treatment, despite other critical RANKL-elicited factors such as NFATc1 being unaffected. MITF may be CSR-inducible [19] although to date we have yet to find strong evidence that stimulating a CSR directly increases MITF mRNA levels in BMM; this may suggest post-transcriptional influences of CSRs such as MITF protein stabilisation. To investigate the possible consequence of elevated MITF levels we studied melphalan effects on transcriptional targets of MITF. These include Dc-stamp which with Oc-stamp critically drives osteoclast cell fusion, a distinctive feature of osteoclasts that increases their resorption pit depth [20, 21]. mRNA levels of both Dc-stamp and Oc-stamp were enhanced by melphalan. Consistent with this, melphalan enhanced cell fusion both in the presence and absence of RANKL. Thus, melphalan has three effects on osteoclast progenitors that increase their response to RANKL, which may be functionally linked: activation of CSR, increased MITF expression and enhanced cell fusion. Together these provide a strong mechanistic basis to explain the effects of melphalan on bone. These data also suggest that osteoclastic effects of melphalan reported here might also be observed with other cytotoxic drugs that induce a CSR in macrophage/osteoclast lineage cells.

The discovery that melphalan increases osteoclastic bone resorption has a number of important implications. First, the increased osteolysis induced by treatment may compound the bone loss caused by myeloma growth in the skeleton. This could further increase the risk of pathological fracture that is already prevalent in this disease. Secondly, the process of bone resorption itself is known to release growth factors from bone matrix, including TGFβ, which can promote tumor growth in the skeleton and may indirectly effect the behavior of myeloma cells in bone. Finally, the bone resorption caused by melphalan treatment could disrupt the myeloma niches on the endosteal bone surface that maintain myeloma cells in a dormant state [4, 22]. In support of this we have recently demonstrated that osteoclasts, and processes that switch on bone turnover, reactivate dormant tumor cells in the skeleton and can increase tumor growth in bone [5]. However, the reactivation of dormant tumor cells in bone may provide a therapeutic benefit since their subsequent proliferation may render them susceptible to chemotherapy-related cytotoxicity [4].

**Figure 6:**
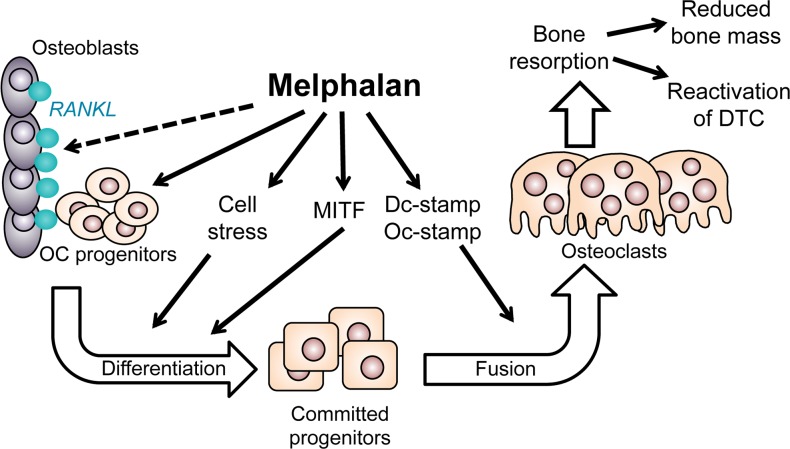
Diagram summarizing the positive effects of melphalan on osteoclast formation in this study Melphalan slightly increased RANKL expression in osteoblastic stromal cells (dotted line); enhanced osteoclast progenitor numbers *in vivo*; increased differentiation *in vitro* at least partly in a cell stress dependent manner; enhanced MITF levels in osteoclast progenitors; and increased cell fusion possibly through increased Dc- and Oc-stamp levels. Increased osteoclast numbers seen both *in vitro* and *in vivo* with mephalan treatment can result in increased bone resorption and thus may potentially also enhance growth of tumor cells or activation of dormant tumors.

Thus, in summary, we have identified pro-osteolytic effects of melphalan treatment and identified a number of molecular mechanisms that maybe responsible for the increase in osteoclastic resorption (Figure 6). These could have important implications for their use in the treatment of patients with myeloma. As a consequence, existing anti-osteolytic therapies such as bisphosphonates should be considered alongside melphalan treatment in order to prevent the negative effects of melphalan treatment on the skeleton.

## MATERIALS AND METHODS

### Reagents and antibodies

Recombinant murine soluble RANKL (RANKL^158-316^–GST fusion protein) was obtained from the Oriental Yeast Co. (Tokyo, Japan) and human M-CSF from Sino Biological Inc. (Beijing, P.R. China). L-cell conditioned media (LCM; a source of secreted murine M-CSF) was prepared as previously described [23]. HSP inhibitor KNK437 were obtained from Sigma-Aldrich (St Louis, MO). Anti-MITF antibody (C5; cat. no. ab12039) was purchased from Abcam (Cambridge, MA). Anti-NFATc1 (cat. no. 556602) was purchased from BD Pharmingen (Franklin Lakes, NJ). Anti-HSP70 (HSPA1A) antibody (cat. no. ADI-SPA-812) was purchased from Enzo Life Sciences (San Diego, CA) and anti-β-actin was obtained from Cell Signaling Technology (Danvers, MA). IgG HRP-conjugated secondary antibodies for immunoblotting were purchased from Thermo Fisher (Scoresby, Victoria, Australia). 1,25 dihydroxyvitamin D_3_ (1,25-D3) was obtained from the Wako Pure Chemical Co. (Tokyo, Japan). TRAP histochemical staining, fast red violet LB Salt, naphthol AS-MX phosphate and dimethylformamaide were purchased from Sigma-Aldrich (Castle Hill, New South Wales, Australia). All other reagent grade chemicals were supplied by Sigma-Aldrich unless noted.

### Animals

8-week-old male C57BL/Kalwrij mice were treated with melphalan (Sigma-Aldrich, 5mg/kg) or vehicle for 3 days (injected intraperitoneally on days 0 and 3), 7 days and 14 days (injected intraperitoneally 3 times a week). Mice were obtained from Australian BioResources (Moss Vale, New South Wales, Australia) and maintained at the Garvan Institute facilities. Procedures were approved by the Garvan Institute / St Vincent's Hospital Animal Ethics Committee in accordance with New South Wales (Australia) State Government legislation.

### Cell lines, cell culture and chemicals

L Cells and RAW264.7 cells were obtained from the American Type Culture Collection (ATCC; Manassas, VA) and maintained in minimal essential medium-α (MEM) containing 10% fetal bovine serum (FBS), 50 units/mL penicillin, 50 mg/mL streptomycin, glutamax supplement and HEPES (all above reagents supplied by Thermo Fisher Scientific). All culture assays utilized this medium (MEM/FBS) unless noted. Primary bone marrow cells for culture were immediately isolated from 6 to 12 week old mice by flushing the bone marrow cavity of the long bones with phosphate buffered saline (PBS). Primary BMM cultures were maintained in 30% L-cell conditioned media to induce BMM proliferation. BMSCs were generated from primary murine bone marrow cells flushed from bisected long bones of mice and maintained in MEM supplemented with 20% FBS. All cells were maintained in a 37°C incubator in a humidified atmosphere containing 5% CO_2_.

### Micro-computed tomography (microCT) of the femur

Bone structure was analyzed using a Skyscan Model 1172 microCT scanner (Bruker, Billerica, MA) at 50 kV, 200 mA with a 0.5-mm aluminium filter using a pixel size of 4.3 mm. Images were captured every 0.4 ° through 180 °, reconstructed and analyzed using NRecon and CTAn software (Bruker). Three-dimensional models and tomographic visualization were created using the Drishti-2 software tool (https://code.google.com/p/drishti-2/) [24].

### Histomorphometric analysis of the femur

Femora were fixed in 4% paraformaldehyde, decalcified in EDTA, embedded in paraffin and 3μm microtome sections (parasagittal plane) cut, dewaxed, reacted for TRAP and counterstained with Mayer's hematoxylin (Sigma-Aldrich). Osteoclast number (Oc.N./BS), and osteoclast surface (Oc.S/BS) were examined in two regions of the bone, the growth plate/primary spongiosa and in mature trabecular bone. For the latter, a region of interest 200 × 200 μm^2^ located 0.4mm below the distal growth plate was employed. These analyses were performed using Osteomeasure histomorphometry software (Osteometrics, Atlanta, GA) [25].

### FACS analysis

Bone marrow cells flushed from long bones were syringed five times through a 23-G needle and filtered through a 100μm nylon cell strainer. Red blood cells were lysed with 0.15 M NH_4_Cl. Cells were resuspended in PBS and filtered through a 70μm strainer. Samples were incubated with Fixable Viability Dye eFluor780 (eBioscience, San Diego, CA) and Fc receptors were blocked with anti-mouse CD16/CD32 (Biolegend, San Diego, CA) at a dilution of 1:100. Cells were then incubated with anti-mouse CD115 APC, anti-mouse CD11b PERCP-Cy5.5, anti-mouse B220 Pe-Cy7, anti-mouse CD11c PE, and anti-mouse Gr-1 Brilliant Violet 421 (BioLegend) at a dilution of 1:100 in PBS/2% FBS. Samples were acquired on a FACSCanto II or LSR II SORP (BD Biosciences, San Jose, CA). Analysis was performed with FlowJo software (Tree Star, Ashland, OR).

### Osteoclast differentiation assays

Osteoclasts were generated from primary BMCs flushed from mouse long bones resuspended in MEM/FBS then added (10^5^ cells/well) to 6mm diameter culture wells. These were stimulated with 20 ng/ml RANKL, plus 25 ng/mL macrophage colony-stimulating factor (M-CSF), and melphalan, KNK437 or vehicle. Osteoclasts were also generated from RAW264.7 cells (5 × 10^3^ per well) in 6mm diameter culture wells in MEM/FBS as above but without M-CSF. For BMC and RAW264.7 osteoclast cultures medium and agents were replaced at day 3, and on day 6 cultures were fixed with 4% paraformaldehyde and histochemically stained for TRAP using a pH 5 phosphate buffered solution containing potassium tartrate, fast red violet LB salt and naphthol AS-MX phosphate as previously described [26]. TRAP-positive MNCs containing 3 or more nuclei were counted as osteoclasts and quantified by inverted light microscopy.

### mRNA expression analysis with qRT-PCR

RNA was extracted from cells using Isolate II RNA mini kit (Bioline, Alexandria, NSW, Australia) according to manufacturer's instructions. RNA (up to 2 μg) was treated to remove genomic DNA with 1 unit of DNase I (New England Biolabs, Beverly, MA) in 10 μL, incubated at 37°C for 10 minutes then DNase inactivated at 75°C. cDNA was synthesized from DNase-treated RNA using a Tetro cDNA synthesis kit (Bioline) according to manufacturer's instructions. The oligonucleotide primers employed in these analyses are listed in Supplementary Table 1. Samples were analyzed in duplicate using a qPCR mix containing 25-50 ng cDNA, 1x iTAQ Sybr Green PCR master mix (Biorad) and 0.2 μM of each primer in a total volume of 10 μL. qPCR reactions were run on ABI Quantstudio7 (Thermo Fisher Scientific) with the following conditions: 10 mins of denaturation at 95°C; 40 cycles of: 95°C (15s), 60°C (60s). To discriminate primer specific amplicon from primer dimers or unspecific PCR products, we also performed melt curve analysis with the following conditions: 95°C (15s), 60°C (60s), 95°C (15s). Comparative (delta-CT) values were calculated using *Gus* as a housekeeping gene, and expressed as relative to the vehicle control.

### Luciferase reporter assays

RAW264.7 cell lines stably transfected with a G418-selected construct that express firefly luciferase driven by promoters containing either NFkB- or an NFAT-response element-containing promoters (NFkB-RAW and NFAT-RAW cells, respectively) were employed in reporter assays as previously described [27]. Briefly, reporter cells were seeded (4×10^4^ cells/6mm diameter culture well) in MEM/FBS, incubated overnight, treatments added in triplicate wells over 24 h then lysed with Passive Lysis Buffer (Promega, Madison, WI) for 24 h at 4°C. Signal was measured using firefly luciferase substrate (Promega) as per manufacturer's instruction using a Clariostar multifunctional microplate reader (BMG Labtech).

### Western blot analysis

Western blot analysis was performed as previously described [18]. Briefly, cell lysates were generated using modified RIPA buffer (50 mM Tris-HCl (pH 7.4), 1% NP40, 0.25% Na deoxycholate, 150mM NaCl) containing phosphatase/protease inhibitor cocktail (Sigma-Aldrich), sonicated and centrifuged. Protein concentrations were determined by BCA protein assay as per manufacturer's instructions (Thermo Fisher Scientific). Cell lysates were run on Criterion 4-20% Tris-Glycine gradient SDS-page electrophoresis gels (Biorad) with Tris-SDS running buffer under reducing conditions and transferred to Immobilon-P polyvinylidene difluoride (PVDF) membranes (Biorad). Membranes were blocked by incubation for 1 h in 3% skimmed milk powder (Diploma brand; Fonterra, Mount Waverley, Victoria, Australia) dissolved in TBS solution containing 0.1% Tween20 detergent (TBST); membranes were then incubated overnight at 4°C with appropriate primary antibodies in TBST/milk. Western blot signal detection was achieved by incubation with appropriate horseradish peroxidase (HRP) conjugated secondary antibodies specific for the primary antibody immunoglobulin species and visualized by ECL detection system (Supersignal West Pico; Thermo Fisher Scientific) according to the manufacturer's instructions. Blots were then imaged with Vilber Lourmat Fusion Fx (Marne-la-Vallée, France) and intensity of bands (normalized to loading control) quantified with ImageJ software (https://imagej.nih.gov/ij/docs/index.html).

### Statistical analysis

Data was analyzed using Prism 6 software (version 6.07; GraphPad, San Diego, CA) and statistical significance determined using unpaired *t*-test and one- and two-way comparisons using ANOVA and Bonferroni's *post hoc* test for multiple comparisons as indicated. Quantitative *in vivo* data is presented as mean ± standard deviation; *in vitro* data is presented as mean ± SEM of three or more pooled independent experiments as specified, and significance is represented graphically by *p< 0.05, **p<0.01 and ***p<0.001 relative to controls.

## SUPPLEMENTARY FIGURES AND TABLE



## Abbreviations

MM: Multiple myeloma
BMC: bone marrow cells
CSR: cell stress response
MITF: microphthalmia-associated transcription factor
NFATc1: nuclear factor of activated T cells 1
Dc-stamp: dendritic cell-specific transmembrane protein
Oc-stamp: osteoclast-stimulatory transmembrane protein
RANKL: receptor activator of NFκB ligand
microCT: microcomputed tomography
BMMs: bone marrow macrophages
qRT-PCR: quantitative RT-PCR
*Atp6v0d2*: vATPase V0d2 subunit
*Ctsk*: Cathepsin-K
Hsp70: heat shock protein 70
HSP90: heat shock protein 90
HSF1: heat shock factor 1
BMSCs: bone marrow stromal cells
1,25-D3: 1,25 dihydroxyvitamin D_3_
TRAP: tartrate resistant acid phosphatase
M-CSF: macrophage colony-stimulating factor
MNCs: multinucleated cells
