# Supplementary material for: Melphalan modifies the bone microenvironment by enhancing osteoclast formation
Source: Oncotarget. 2017 Jul 10;8(40):68047–58. doi: 10.18632/oncotarget.19152 (PMC5620235; doi:10.18632/oncotarget.19152)
Supplement: Supplementary file 1 [file oncotarget-08-68047-s001.pdf]

Melphalan modifies the bone microenvironment by enhancing osteoclast formation

SUPPLEMENTARY MATERIALS

Osteoblast differentiation assay

Osteoblasts were generated from primary murine bone marrow cells cultured in MEM containing 20% FBS in 16 mm diameter culture wells ($3x10^6$ cells/well) expanded for 4 days or until 80% confluent. MEM/FBS containing osteoblast differentiating factors (50 µg/ml ascorbic acid, 2.5 µM dexamethasone and 10 mM β-glycerolphosphate; Sigma-Aldrich) was added and changed every 3 days until day 21. Cells were washed with PBS and fixed with 4% paraformaldehyde for 15 mins, rinsed and stained with 0.5% Alizarin Red (Sigma Aldrich) in water for 30 mins, washed, dried and images of the plates taken with an flat-bed scanner (model v800, Epson, North Ryde, NSW Australia). Alizarin red was then eluted with 10% cetyl pyridinium chloride (CTP; Sigma-Aldrich) in PBS overnight and quantified by measuring 562 nm absorbance (Clariostar plate reader, BMG Labtech, Offenburg, Germany) relative to standard alizarin red solutions.

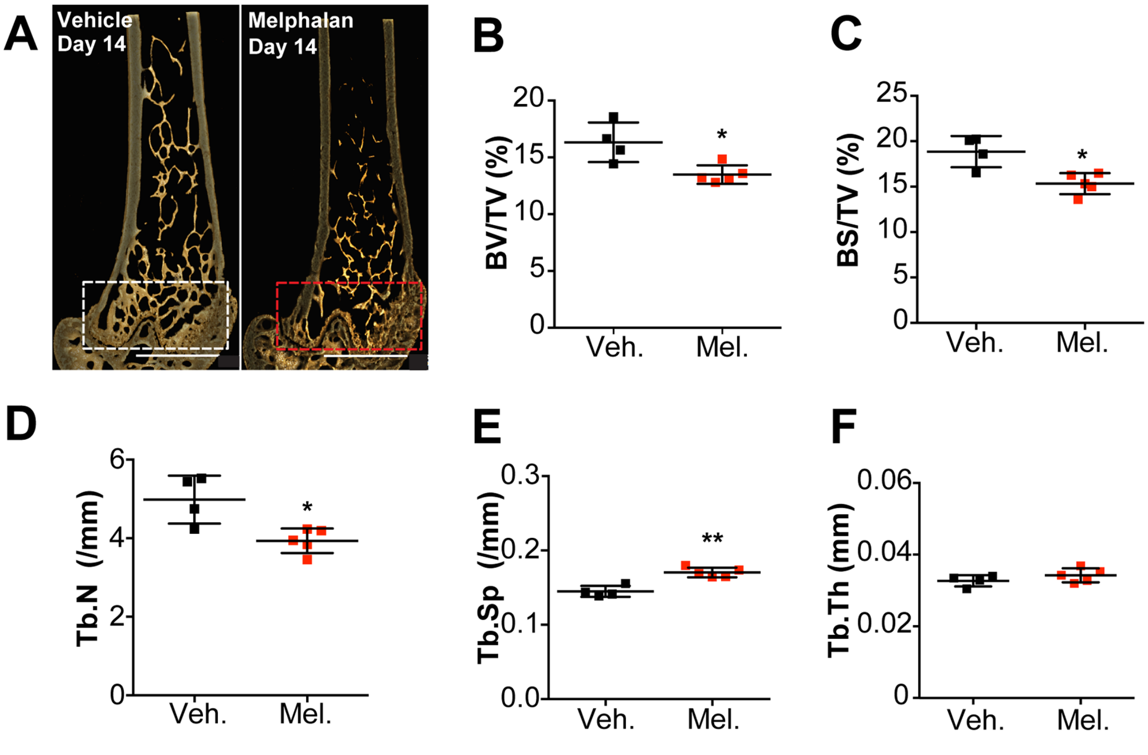

Supplementary Figure 1: Melphalan treatment reduces bone mass at the growth plate and primary spongiosa after 14 days. MicroCT analysis bone in the growth plate and primary spongiosa of the femora of mice treated with vehicle (Veh.) or melphalan (Mel.) for 14 days, including **(A)** Three-dimensional reconstructions of microCT scans of representative femora of mice treated with vehicle or melphalan for 14 days as indicated (region of interest for analysis are indicated by white or red box; scale bar = 1mm), **(B)** bone volume/total volume (BV/TV), **(C)** bone surface area (BS/TV), **(D)** trabecular number (Tb.N), **(E)** trabecular separation (Tb.Sp) and **(F)** trabecular thickness (Tb.Th). Numerical data presented as individual data points with indicated mean ± SD and represent 4-5 mice per group. *p<0.05; **p<0.01; ***p<0.001 using Student's t-test.

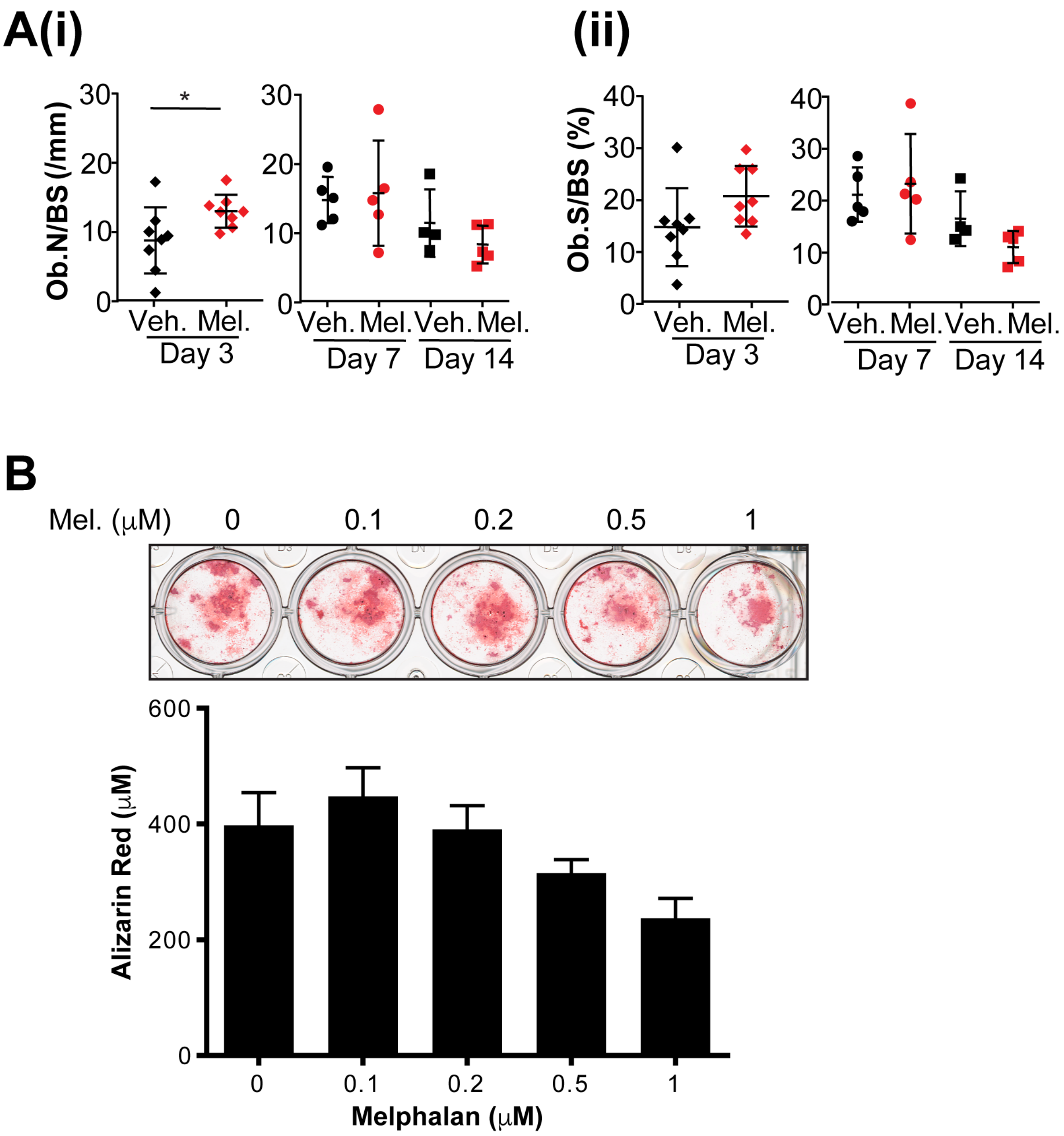

Supplementary Figure 2: Melphalan treatment has minimal effect on osteoblast *in vivo* and *in vitro*. **(A)** Quantification by histomorphometry of (i) osteoblast numbers and (ii) bone surface covered by osteoblasts in the trabecular bone of secondary spongiosa of femora. Numerical data presented as individual data points with indicated mean ± standard deviation and represent 5-8 mice per group. *$p<0.05$; **$p<0.01$; ***$p<0.001$ using ANOVA/Dunnett's post hoc test or (for two-way comparisons) t-tests. **(B)** Photomicrographs of mineralization assay with primary bone marrow stromal cells treated with vehicle and different sublethal doses of melphalan for 21 days in the presence of osteoblastic differentiation factor and the quantification of eluted alizarin red stain of mineralization from 4 independent experiments (mean ± S.E.M).

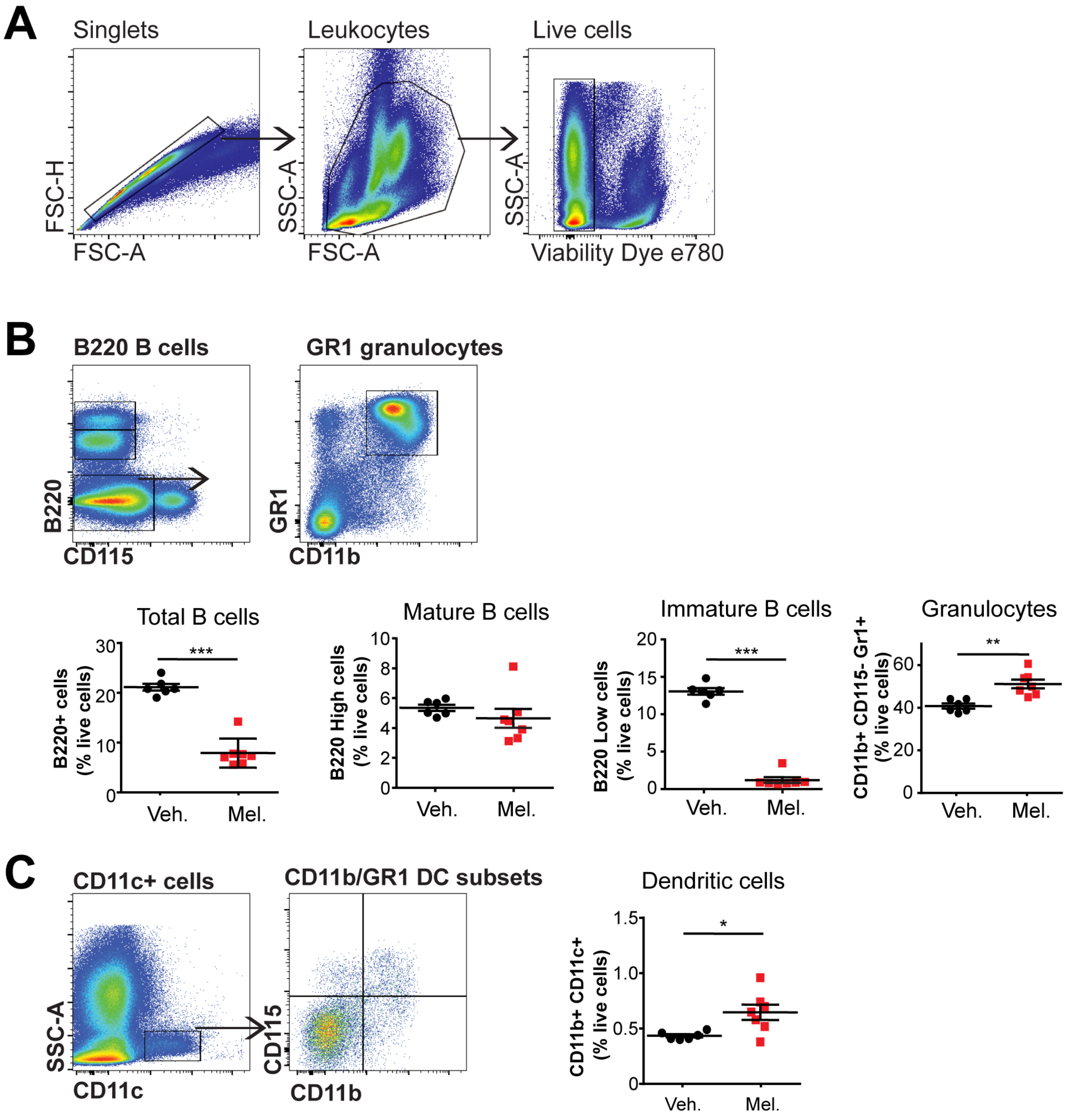

Supplementary Figure 3: Additional bone marrow populations affected by 3 days of melphalan treatment. (A) Total live cells were analyzed as a proportion of all cells. Live cells were then examined for **(B)** B cell populations (B220+), granulocytes and **(C)** dendritic cells (CD11b+CD11c+). Numerical data presented as individual data points with indicated mean ± S.E.M and represent 8 mice per group. *p<0.05; **p<0.01; ***p<0.001 using Student's t-test.

Supplementary Table 1: Sequences of quantitative real time polymerase chain reaction (qRT-PCR) primers

Dc-stamp

forward primer: 5’-CTAGCTGGCTGGACTTCATCC-3’

reverse primer: 5’- TCATGCTGTCTAGGAGACCTC-3’

Oc-stamp

forward primer: 5’- TGGGCCTCCATATGACCTCGAGTAG-3’

reverse primer: 5’- TCAAAGGCTTGTAAATTGGAGGAGT-3’

Acp5

forward primer: 5’-GGGGACAATTTCTACTTCACTGG-3’

reverse primer: 5’-GCAAACGGTAGTAAGGGCTG-3’

Atp6v0d2

forward primer: 5’- AAGCCTTTGTTTGACGCTGT-3’

reverse primer: 5’- AAGCCTTTGTTTGACGCTGT-3’

Ctsk

forward primer: 5’-GGCCAGTGTGGTTCCTGTT-3’

reverse primer: 5’-CAGTGGTCATATAGCCGCCTC-3’

Nfatc1

forward primer: 5’-AGCCCATCCTTGCCTGCCCT-3’

reverse primer: 5’-CCGTGTAGCTGCACAATGGGG-3’

Gus

forward primer: 5’-GACTGGCCGCTGCTGGTAAA-3’

reverse primer: 5’-GTAGGGATAGTGGCTGGTACGAAA-3’
